# Supplementary material for: LncPLAAT3-AS Regulates PLAAT3-Mediated Adipocyte Differentiation and Lipogenesis in Pigs through miR-503-5p
Source: Genes (Basel). 2023 Jan 6;14(1):161. doi: 10.3390/genes14010161 (PMC9859061; doi:10.3390/genes14010161)
Supplement: Supplementary file 1 [file genes-14-00161-s001.zip › Supplementary Figure.pdf]

A

Group	Sample	Clean Reads (Milions)	Q20 (%)	Q30 (%)	Clean Bases (G)	Total Mapped (%)
Overespression group	LncRNA	44.72	95.71	88.13	6.71	90.72
	LncRNA	44.89	95.88	88.61	6.73	90.79
	LncRNA	44.92	95.73	88.19	6.74	90.84
Inhibitor group	siRNA	44.67	95.59	87.86	6.70	90.37
	siRNA	44.66	95.64	88.01	6.70	90.26
	siRNA	44.77	95.97	88.86	6.72	91.20
Inhibitor NC group	Control	44.82	97.86	93.28	6.72	94.67
	Control	44.95	97.79	93.08	6.74	94.57
	Control	44.82	97.81	93.14	6.72	94.51

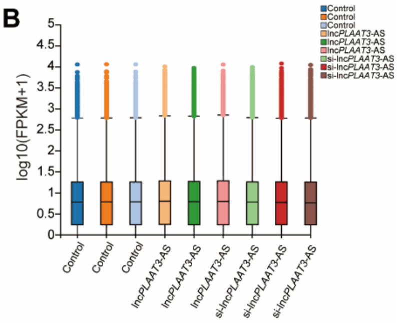

Figure S1 (**A**) mRNA sequencing data reads quality statistics; (**B**) Boxplot of mRNA sequencing expression in transfected cells.

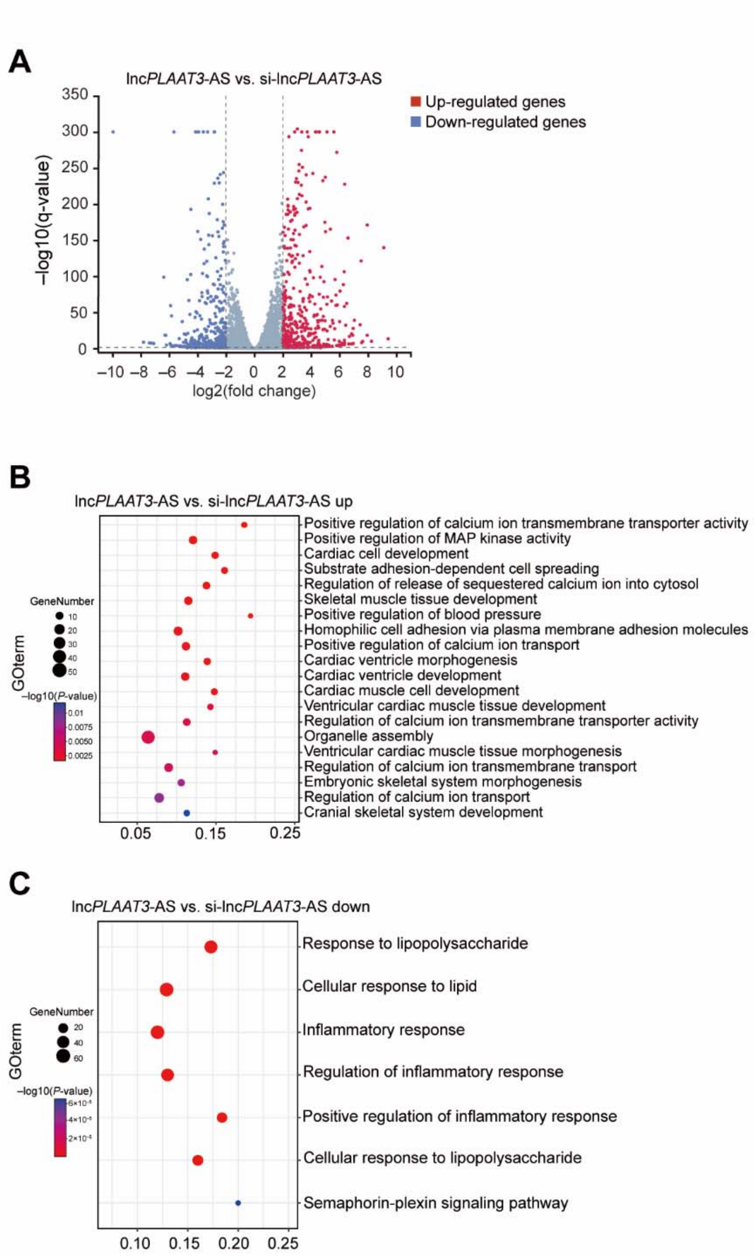

Figure S2 (**A**) Differentially expressed gene volcano plot; (**B~C**) The top 20 KEGG pathways of differential genes. Sequentially for lncPLAAT3-AS vs si-lncPLAAT3-AS differential genes with up-regulated expression, lncPLAAT3-AS vs si-lncPLAAT3-AS differential genes with down-regulated expression.

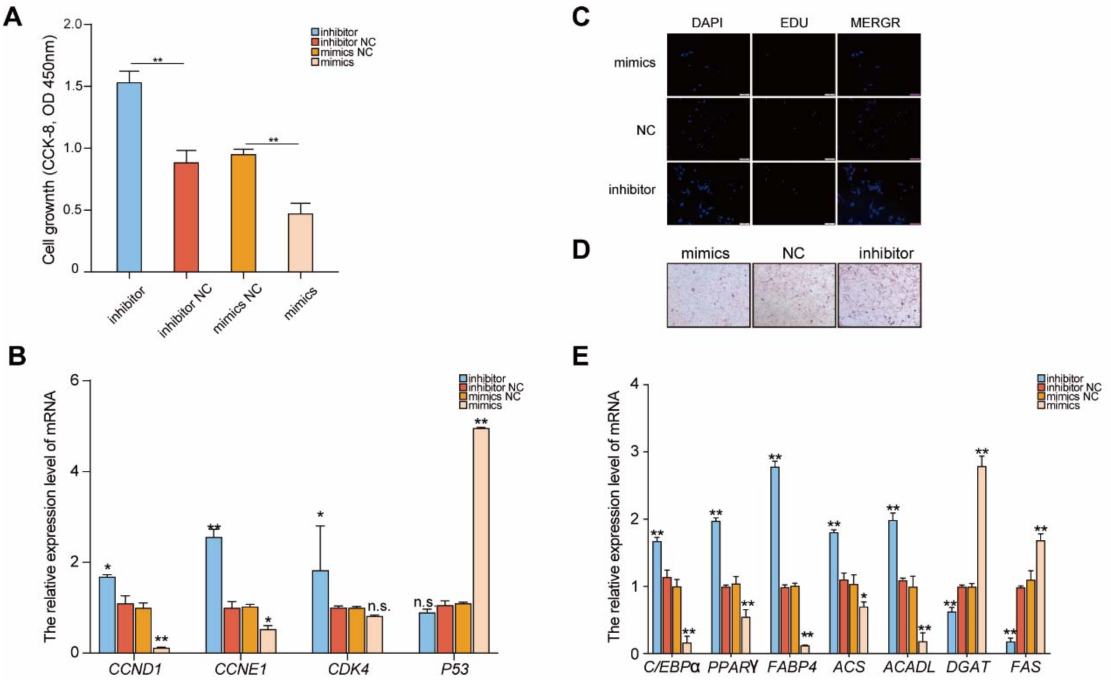

Figure S3 (**A**) cell proliferation was evaluated at 24 h of proliferation by performing CCK-8, miR-503-5p mimic negative control for miR-503-5p mimic NC, miR-503-5p inhibitor negative control for miR-503-5p inhibitor NC; (**B**) qRT–PCR analysis of genes related with cell cycle; (**C**) Representative images illustrating the EdU of PK15 cells to transfected with miRNA-503-5p mimics or miRNA-503-5p inhibitor; (**D**) Cells were stained with oil red O,NC is negative control; (**E**) The expression levels of gene-related adipogenic, fatty acid oxidationin and fatty acid transportation synthesis were measured by qRT-PCR. Scal bars 50μm. All results are presented as means ± SEM (n = 3). * $P < 0.05$; ** $P < 0.01$, n.s. no significant difference.

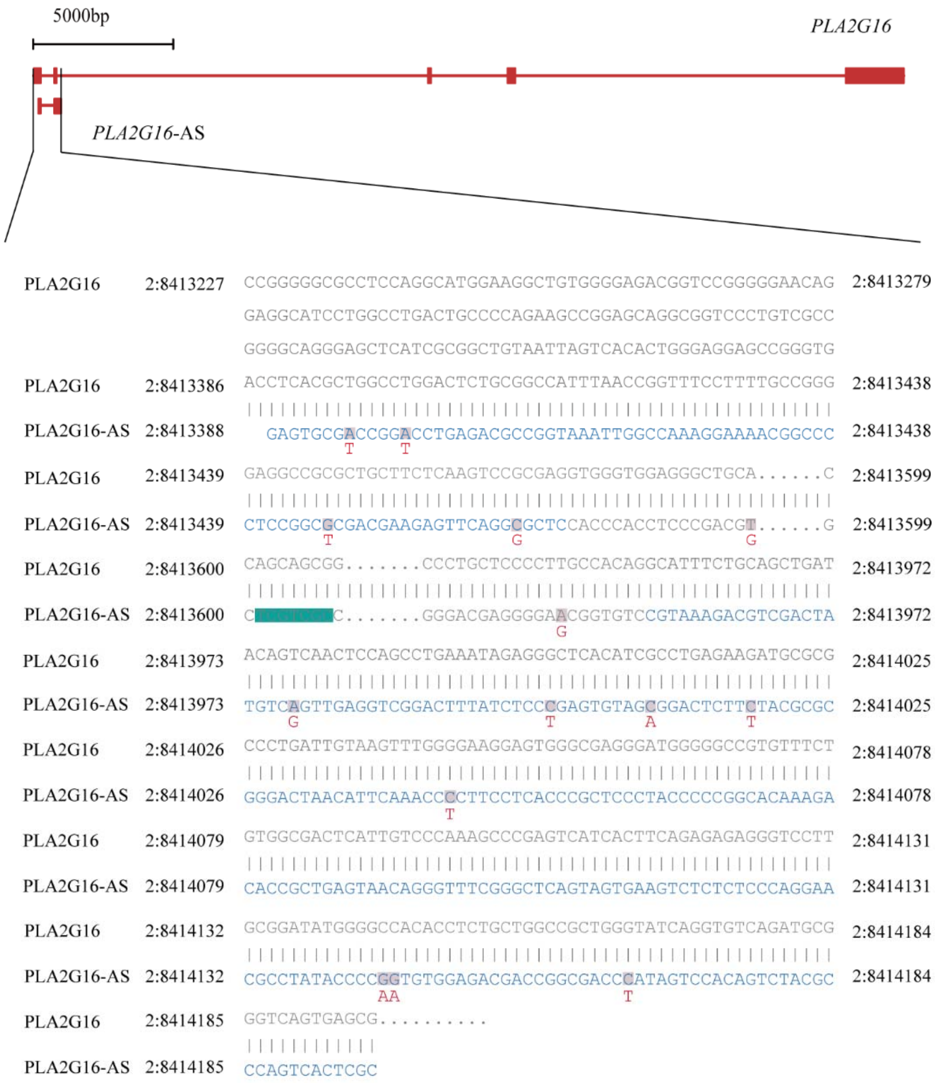

Figure S4 The single nucleotide mutation site of lnc*PLAAT3*-AS SNP, red is the mutated base, and the green area is the binding region of lnc*PLAAT3*-AS and miR-503-5p. We used ISWINE database (http://iswine.iomics.pro/pigdna/dna/index?access_token=null&lang=en_US) to predict the biological effect of the significant SNPs on lncRNAs.

.
